# Exposure to elevated glucose concentrations alters the metabolomic profile of bovine blastocysts

**DOI:** 10.1371/journal.pone.0199310

**Published:** 2018-06-20

**Authors:** Karen Uhde, Helena T. A. van Tol, Tom A. E. Stout, Bernard A. J. Roelen

**Affiliations:** 1 Department of Farm Animal Health, Faculty of Veterinary Medicine, Utrecht University, Utrecht, The Netherlands; 2 Department of Equine Sciences, Faculty of Veterinary Medicine, Utrecht University, The Netherlands; Justus Liebig Universitat Giessen, GERMANY

## Abstract

Chronically high blood glucose concentrations are a characteristic of diabetes mellitus. Maternal diabetes affects the metabolism of early embryos and can cause a delay in development. To mimic maternal diabetes, bovine *in vitro* fertilization and embryo culture were performed in fertilization medium and culture medium containing 0.5, 2, 3, and 5 mM, glucose whereas under control conditions, the medium was glucose free (0 mM). Compared to control conditions (0 mM, 31%), blastocyst development was decreased to 23% with 0.5 and 2 mM glucose. Presence of 3 or 5 mM glucose in the medium resulted in decreased blastocyst rates (20% and 10% respectively). The metabolomic profile of resulting day 8 blastocysts was analysed by UPLC-MS/MS, and compared to that of blastocysts cultured in control conditions. Elevated glucose concentrations stimulated an increase in glycolysis and activity of the hexosamine pathway, which is involved in protein glycosylation. However, components of the tricarboxylic acid cycle, such as citrate and alpha-ketoglutarate, were reduced in glucose stimulated blastocysts, suggesting that energy production from pyruvate was inefficient. On the other hand, activity of the polyol pathway, an alternative route to energy generation, was increased. In short, cattle embryos exposed to elevated glucose concentrations during early development showed changes in their metabolomic profile consistent with the expectations of exposure to diabetic conditions.

## Introduction

In diabetic patients, glucose utilization and storage is not regulated properly, resulting in hyperglycemia and glycosuria. Of the two major types of diabetes, type 1 is an autoimmune disease that generally onsets during childhood, whereas type 2 diabetes is the result of insulin resistance, and is mostly triggered by an unhealthy lifestyle, including poor nutrition and physical inactivity. Diabetes is an increasingly prevalent disease; in 2015, it was estimated that 415 million people worldwide suffered from some form of diabetes, and it is expected that this will increase to 642 million people by 2040, largely as a result of an increasing incidence of insulin resistance linked to obesity [1]. In addition to diabetes-related pathology in the primary patient, there is an increased risk of gestational complications, including spontaneous abortion and the development of metabolic diseases in the children of mothers who are, or become, diabetic during pregnancy [2]. Gestational diabetes occurs spontaneously and is triggered by pregnancy-related changes in the endocrine environment [3], but disappears after birth of the child. However, women that develop gestational diabetes, and their children, have a higher risk of developing type 2 diabetes later in life [1]. In 2015, it was estimated that 20.9 million children were born to mothers suffering from hyperglycemia during pregnancy [1].

Although blood glucose concentrations fluctuate markedly in healthy individuals depending primarily on the time and composition of their last meal, they are homeostatically regulated by insulin, glucagon and other catabolic hormones, such that they return to normoglycemic resting values between approximately 4 and 6 mM. By contrast, the blood glucose concentrations of diabetic patients are chronically elevated to approximately 12–19 mM, primarily as a result of either inadequate insulin production (type 1 diabetes) or insensitivity to the actions of insulin (type 2 diabetes). In women, high blood glucose concentrations are reflected in reproductive tract fluids, and may therefore affect oocyte maturation and early embryo development [4]. In cows the glucose concentrations vary between 0.05 and 4.5 mM in the reproductive tract, which is lower compared to the glucose concentrations detected in blood (5.8–7.7 mM) [5–8]. Indeed, careful regulation of the metabolic status of women suffering from diabetes during pregnancy has been shown to decrease the incidence of fetal abnormalities [9]. How, and to what extent, high glucose concentrations affect pre-implantation development is less well understood, primarily because of the relative inaccessibility of affected early embryos. To investigate the possible effects of maternal diabetes on oocytes or early embryos, various animal models have therefore been developed. A diabetic mouse model demonstrated that oocytes from hyperglycemic animals are smaller and exhibit delayed meiotic maturation when compared to oocytes from control mice [10, 11]. In addition, both oocyte developmental competence [12–14] and early embryonic development are compromised by hyperglycemia in diabetic animal models including the mouse, rat, rabbit, sheep and cow [4, 15–19]. While most diabetes studies have been performed in rodent models, using bovine blastocysts has several advantages for extrapolation to human development. The main advantages of bovine oocytes include accessibility and avoiding the use of experimental animals, since cow ovaries can be obtained as a by-product from slaughterhouses. Furthermore, like man, the cow is a mono-ovulatory species with a similar time course of oogenesis and folliculogenesis, making the cow a good animal model for human reproduction [20, 21]. In addition, germinal vesicle breakdown during maturation requires protein synthesis in human and bovine oocytes [22, 23], which is not needed in the mouse [24].

During oocyte maturation, the cumulus cells that surround the oocyte are glycolytically active; they take up glucose and generate pyruvate, the preferred metabolic substrate of the oocyte [25–27] and preimplantation embryo [28, 29]. In addition, the pentose phosphate pathway is reported to play an important role in glucose utilization during meiotic maturation [30, 31] and early embryo development [32, 33]. Overall, it is thought that, before it enters the uterus, the embryo’s main energy substrates are pyruvate and lactate, which are metabolized via oxidative phosphorylation. After arrival in the relatively anaerobic environment of the uterus, the embryo switches its primary mode of metabolism to glycolysis [34].

Exactly how high glucose concentrations affect the metabolism of the preimplantation embryo is not well understood. Here, we used bovine *in vitro* fertilization and embryo development both in the presence of exogenous glucose, to create an environment mimicking that expected in a diabetic mother, and compared the metabolomic profile of resulting blastocysts and the medium conditioned by embryo culture with those of glucose-free culture.

## Materials and methods

All chemicals were purchased from Sigma-Aldrich (St. Louis, MO, USA) unless otherwise stated.

### Collection of cumulus-oocyte complexes, *in vitro* maturation, fertilization and embryo culture

Cattle ovaries were collected from a local slaughterhouse (Gosschalk, Epe, The Netherlands) and transported to the laboratory in a thermos flask. On arrival at the laboratory, the ovaries were rinsed in tap water and maintained at 30°C in physiological saline (0.9%) containing 100 IU/ml penicillin and 100 μg/ml streptomycin. Cumulus-oocyte complexes (COCs) were then aspirated from small antral follicles (2–8 mm) using an 18-gauge needle connected via a 50 ml collection tube to a low-pressure aspiration pump. COCs with several cumulus cell layers were selected and, after washing in HEPES buffered M199 (Gibco BRL, Paisley, UK), were matured *in vitro* in groups of 50–60 COCs. All culture steps (maturation, fertilization and embryo culture) took place in 4-well culture plates (Nunc A/S, Roskilde, Denmark). To allow *in vitro* maturation, COCs were incubated for 23h in 500 μl NaHCO_3_-buffered M199 (Gibco BRL), which contains 5.5 mM glucose, supplemented with 1% penicillin-streptomycin (Gibco BRL), 0.02 IU FSH/ml (Sioux Biochemical Inc., Sioux Centre IA, USA), 0.02 IU LH/ml (Sioux Biochemical Inc.), 7.7 μg/ml cysteamine and 10 ng/ml epidermal growth factor, at 39°C in a humidified atmosphere containing 5% CO_2_.

*In vitro* fertilization was performed as described previously [6, 35]. In brief, fertilization medium which does not contain glucose but instead contains sodium pyruvate as an energy source, was supplemented with 1.8 IU/ml heparin, 20 μM d-penicillamine, 10 μM hypotaurine, and 1 μM epinephrine. Frozen-thawed spermatozoa from an *in vitro* fertility proven bull was washed through a Percoll gradient and used at a concentration of 1×10^6^ sperm cells/ml. After sperm-oocyte co-incubation for 18-22h at 39°C in a humidified atmosphere containing 5% CO_2_ and 7% O_2_, presumptive zygotes were denuded of their cumulus investment by vortexing for 3 min, and transferred to 500 μl synthetic oviductal fluid (SOF) [36], containing sodium pyruvate, BSA and sodium lactate, for further culture. Assessment of presumptive embryos for cleavage was performed on day 5 of culture, and only cleaved embryos were transferred to fresh SOF and cultured for an additional 3 days (until day 8), when blastocyst development was assessed. To determine an appropriate glucose concentration for the metabolome analysis, fertilization medium and SOF were supplemented with 0.5, 2, 3, and 5 mM glucose. Control conditions were without glucose. Based on embryo development data, 3 mM glucose and no glucose (control) were used for fertilization medium and SOF for the definitive metabolome analysis.

### Collection of blastocysts and culture medium for metabolomic analysis

Day 8 blastocysts, which did not show any morphological abnormalities, were washed in PBS, snap frozen in liquid nitrogen and stored at -80°C until processing for metabolomic analysis. In total, 3 samples from each condition, containing between 538 and 590 blastocysts per sample were analysed. Medium (500 μl) was conditioned by incubation of at least 50 embryos with more than eight-cells from day 5 to day 8 in SOF medium, either without glucose (control) or supplemented with 3 mM glucose.

### Sample preparation and metabolomic analysis

Sample preparation and ultra-high performance liquid chromatography-tandem mass spectroscopy (UPLC-MS/MS) were performed as described previously [37] by Metabolon Inc. (Durham, NC USA). In brief, 4 analysing methods were used, two separate reverse phase (RP) UPLC-MS/MS methods employing positive ion mode electrospray ionization, one analysis by RP/UPLC-MS/MS with negative ion mode electrospray ionization, and one analysis by hydrophilic interaction chromatography (HILIC)/UPLC-MS/MS with negative ion mode electrospray ionization. Raw data were extracted, peak-identified and QC-processed using Metabolon’s proprietary hardware and software. Measurements for blastocysts were normalized against total protein concentration. In the case of missing values, imputation was used to calculate the fold change (marked with an asterisk in the supplementary tables).

### Statistical analysis

Statistical analysis of the success of blastocyst development in medium with different glucose concentrations was performed using SigmaPlot software (Systat software Inc., San Jose, CA, USA), these data are expressed as means ± SD. To compare the percentage of blastocysts between treatment groups, after testing for normality of distribution, an analysis of variance (ANOVA) was performed followed by a post-hoc Bonferroni test. Welch’s two-sample t-test was used to compare metabolite concentrations between 0 and 3mM glucose conditions. A P-value < 0.05 was considered to be statistically significant.

## Results

**Fig 1:**
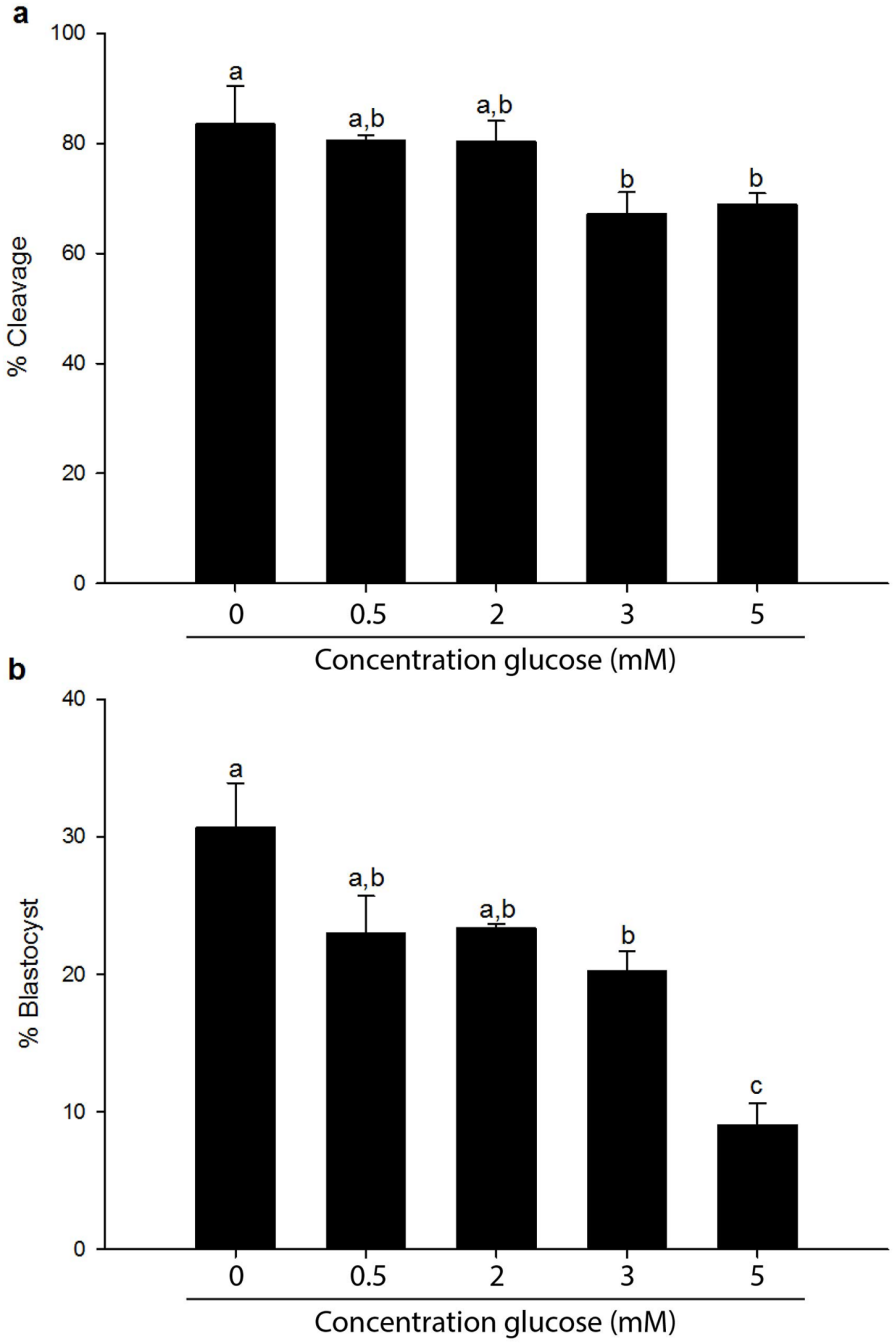
Effect of glucose on bovine cleavage and blastocyst development. Fertilization and embryo culture was performed in culture medium without supplementary glucose (control) and in medium containing final glucose concentrations as indicated. A) Cleavage was evaluated on day 5 of post-fertilization culture. B) Blastocyst development was evaluated on day 8 of post-fertilization culture. Data are depicted as mean ± SD; columns with different letters differ significantly, p < 0.05.

In order to determine tolerance of bovine embryos to glucose, bovine oocytes were fertilized and cultured to the blastocyst stage in the presence of exogenous glucose. Under standard conditions fertilization medium and synthetic oviductal fluid (SOF) do not contain glucose (0 mM). To find an optimal glucose concentration for stimulation, concentrations between 0.5 mM and 5 mM glucose were tested. Cleavage was compromised at glucose concentration of 3 mM (67%) and 5 mM (68%), compared to 83% under control conditions (Fig 1A). Presence of 0.5 mM and 2 mM glucose resulted in 80% cleavage. Comparison of blastocyst development showed a decrease to 23% in the presence of 0.5 or 2 mM glucose, compared to 31% in the control group. Presence of 3 mM glucose resulted in more reduced blastocyst rates (20% and 10%, respectively: Fig 1B). For further experiments 3 mM glucose was chosen, because it showed a clear compromise on embryo development, while still providing adequate blastocyst development to collect blastocysts for the metabolome analysis.

The percentages of oocytes that developed to blastocysts decreased significantly at glucose concentrations as low as 3 mM (Fig 1B). To further understand how blastocysts that developed in the presence of elevated glucose concentrations differed to those that developed in the absence of exogenous glucose, the metabolomic profiles of the blastocysts were determined by mass spectrometry. In total, 1719 control blastocysts and 1698 glucose-stimulated (3 mM) blastocysts were divided into three groups for metabolite identification. In addition, the medium in which embryos had been cultured from days 5 to 8 was analysed for metabolite concentrations.

**Fig 2:**
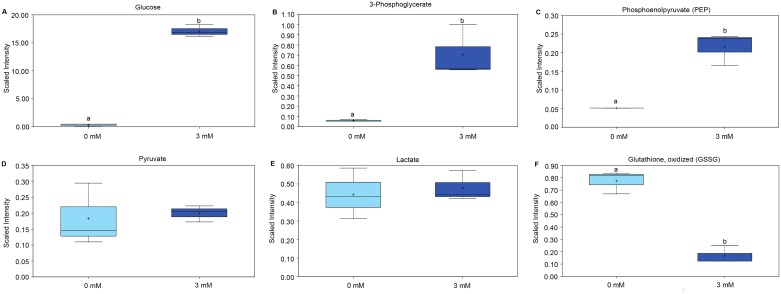
Selected biochemicals detected in bovine blastocysts. Box and whisker plots for biochemical components detected in day 8 blastocysts produced in medium in the presence or absence (control) of 3 mM glucose. A) glucose (p = 0.0396), B) 3-phosphoglycerate (p = 0.0015), C) phosphoenolpyruvate (p = 0.0077), D) pyruvate (p = 0.6239), E) lactate (p = 0.6635) and F) oxidized glutathione (GSSG) (p = 0.0146). The upper whiskers represent the maximum, and the lower whiskers the minimum values. The plus-signs indicate the mean values, while the median values are represented by a black line within the boxes. Boxes with different letters differ significantly. Light blue—d8 blastocysts without supplementary glucose; blue—d8 blastocysts cultured in the presence of 3 mM glucose.

**Fig 3:**
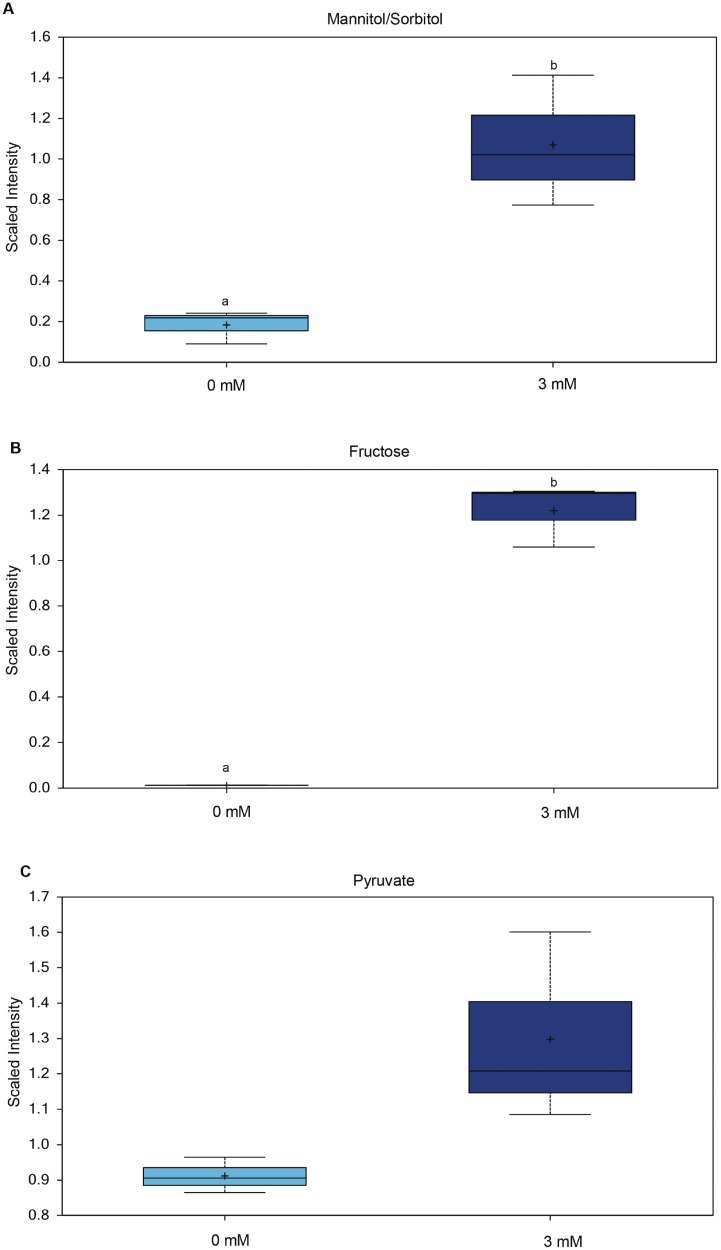
Selected biochemicals detected in bovine embryo conditioned medium. Box plots for biochemical components detected in SOF medium conditioned by bovine embryos with more than eight cells cultured for 3 days (day 5 –day 8 of *in vitro* culture). A) mannitol/sorbitol (p = 0.0131), B) fructose (p = 0.0002) and C) pyruvate (p = 0.0902). The upper whiskers represent the maximum, and the lower whiskers the minimum values. The plus-signs indicate the mean values, while the median values are represented by a black line within the boxes. Boxes with different letters differ significantly. Light blue—conditioned glucose-free medium; blue—conditioned 3 mM glucose supplemented medium.

The metabolomic profiles revealed 22 biochemical constituents that increased and 41 that decreased significantly in concentration, in glucose-stimulated compared to control blastocysts. The comparison of conditioned media from the two types of culture showed significantly altered concentrations of 18 biochemical compounds, 11 of which were increased and 7 decreased when blastocysts were cultured in medium containing elevated glucose concentrations. As expected, the intraembryonic glucose concentration increased markedly when embryos were cultured in the presence of 3 mM glucose (Fig 2A, S1 Table). Intracellular concentrations of 3-phosphoglycerate and phosphoenolpyruvate (Fig 2B and 2C, S1 Table) were also increased significantly, indicating enhanced glycolytic activity; however, intracellular concentrations of pyruvate and lactate were not altered (Fig 2D and 2E, S1 Table). Embryonic mannitol/sorbitol and fructose concentrations were however increased, indicating glucose metabolism via the polyol pathway (S1 Table). Surprisingly, given that sorbitol does not diffuse efficiently through the plasma membrane, the concentrations of sorbitol and fructose were also increased in the medium conditioned by glucose-stimulated embryos (Fig 3A and 3B, S2 Table). And while the pyruvate concentrations in the conditioned medium tended to increase (Fig 3C, S2 Table), the increase did not reach statistical significance (P = 0.09). Increased intracellular concentrations of N-acetylglucosamine, N-acetyl-glucosamine 1-phosphate, UDP-N-acetylglucosamine/galactosamine, and N-acetylneuraminate (S1 Table) indicate increased hexosamine pathway activity in the glucose-stimulated blastocysts. Sedoheptulose, an intermediate of the pentose phosphate pathway, was detected in glucose-stimulated blastocysts only. A reduced concentration of oxidized glutathione was also detected in glucose-stimulated blastocysts (Fig 2F).

## Discussion

Metabolic stress induced by supraphysiological glucose concentrations has previously been reported to alter gene expression in preimplantation embryos [4, 19]. Furthermore, high maternal blood glucose concentrations are thought to compromise embryo development and predispose to gestational complications. In this study, we investigated changes in the metabolomic profile of bovine blastocysts resulting after culture in glucose-supplemented (3 mM) fertilization and embryo culture media in comparison to blastocysts produced in control media (0 mM).

The glucose concentrations reported for bovine oviductal fluid vary between 0.05 and 4.5 mM and are lower than those detected in blood (between 5.8 and 7.7 mM) [5–8]. In this respect, while it is not too surprising that a glucose concentration of 10 mM appears to be lethal to bovine embryos during *in vitro* culture [19], it is surprising that concentrations of 4.5 mM [38] or 5 mM [19] compromised embryo development, even when elevated glucose was only present during part of the embryo culture period and especially when the glucose was present early in the culture period. In the current study, elevated glucose concentrations (3 mM) were present throughout fertilization and embryo culture up to the blastocyst stage. Although the percentage of blastocysts that developed from oocytes in the presence of 3 mM glucose was reduced, the blastocysts formed appeared otherwise grossly normal and were used for metabolomic analysis.

Early embryos, in the first few days after fertilization, have been reported to primarily utilize pyruvate as an energy substrate [39, 40]; it is only after compaction at the morula stage that glucose becomes an important substrate, via glycolysis, for energy production and lipid biosynthesis. In addition, glucose can enter the pentose phosphate pathway and be used for nucleotide synthesis, NADPH production and regulation of the intracellular redox status [41]. The increased levels of 3-phosphoglycerate and phosphoenolpyruvate in embryos exposed to higher concentrations of glucose indicate increased glycolysis under these conditions. Additionally, the 25-fold increase in sedoheptulose indicates that glucose is also channelled into the pentose phosphate pathway. Glucose-exposed blastocysts also exhibited a 310-fold increase in mannitol/sorbitol concentrations. In this respect, the polyol pathway converts glucose into sorbitol, which is further converted into fructose; increased sorbitol concentrations therefore indicate increased activation of the polyol pathway to generate energy. The increased concentrations of mannitol/sorbitol in the blastocyst conditioned medium were less expected, because of the poor diffusion of sorbitol through the plasma membrane. On the other hand, sorbitol accumulation in cells is a common feature of diabetes, and leads to accumulation of reactive oxygen species (ROS). The release of sorbitol into the medium might counteract this ROS accumulation in the blastocysts.

Increased N-acetylglucosamine 1-phosphate and UDP-N-acetylglucosamine concentrations suggest engagement of the hexosamine pathway in blastocysts encountering elevated glucose concentrations. In this respect, it has been reported that protein glycosylation via the hexosamine pathway is the underlying mechanism for the embryotoxic effect of excess glucose [42]. It has also been suggested that activation of the hexosamine pathway leads to increased TGF-beta-1 expression [43, 44]. However, the transcriptome of glucose-exposed bovine embryos did not show a change in *TGFB1* mRNA expression, although Ingenuity Pathway Analysis did indicate enhanced TGF-beta signalling [19].

Hyperglycemic conditions lead to the production of reactive oxygen species, which can stimulate the polyol pathway by inhibiting glyceraldehyde-3-phosphate dehydrogenase activity [44]. It has therefore been suggested that hyperglycemic conditions can trigger the Warburg effect in bovine embryos, in particular anaerobic glycolysis and lactate production [19]. Although pyruvate oxidation via the tricarboxylic acid cycle seemed to be reduced, which is a characteristic of the Warburg effect, we did not observe an increase in intra- or extracellular lactate production by embryos cultured in 3 mM glucose.

Blastocysts stimulated with glucose showed a reduction in oxidized glutathione concentrations, which is also reported to occur under diabetic conditions [45]. Glutathione is a tripeptide synthetized from glycine, cysteine and glutamate. Two of the amino acids, glycine and cysteine were decreased in glucose treated blastocysts, which hints at decreased glutathione synthesis. Patients with uncontrolled type 2 diabetes also showed a decrease in concentrations of the amino acids glycine and cysteine [46]; the lowered levels of glutathione might be a reason for higher levels of oxidative stress during diabetes.

**Fig 4:**
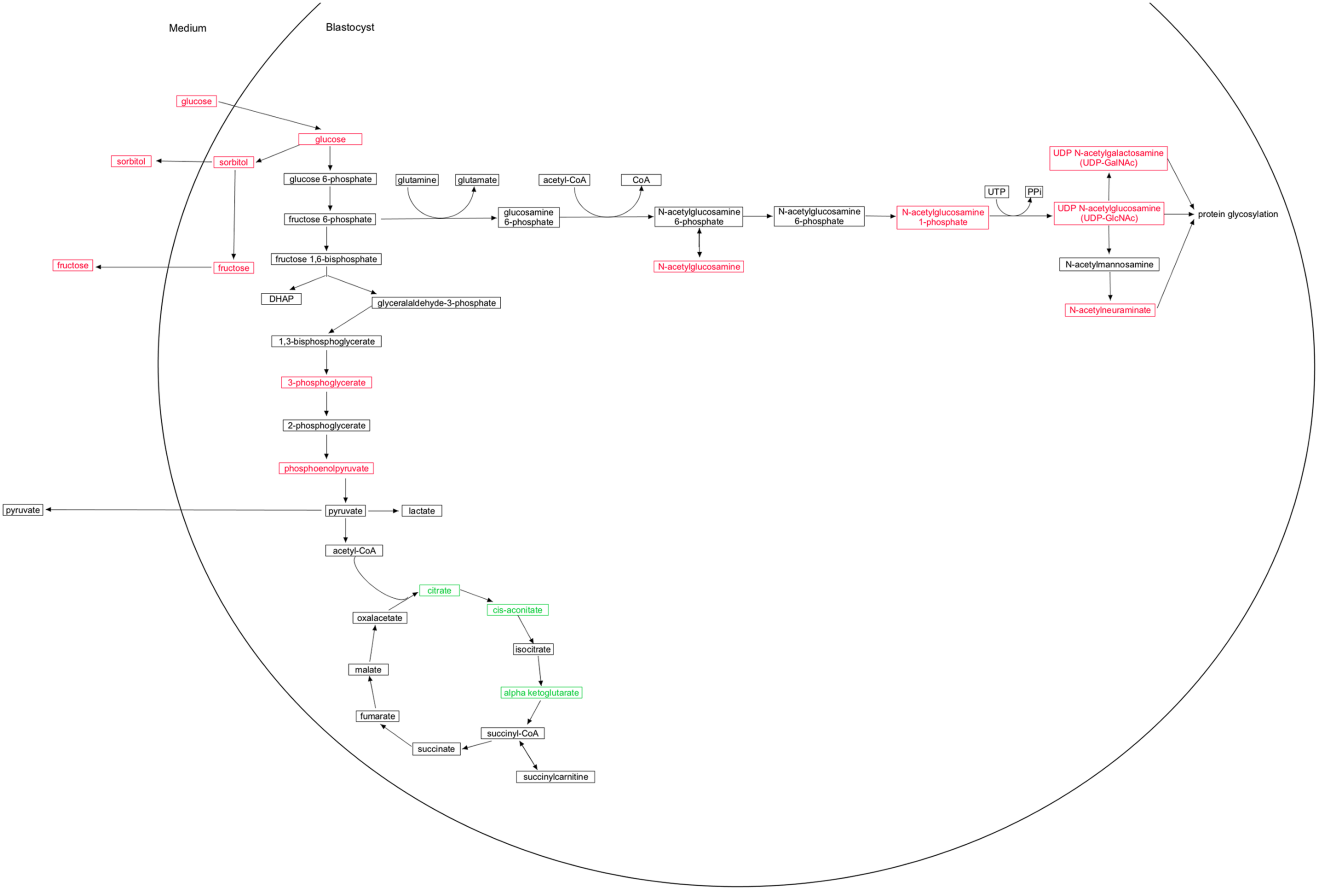
Schematic diagram of biochemicals in bovine blastocysts affected by glucose exposure. Biochemical components of glycolysis, the tricarboxylic acid cycle, and the hexosamine pathway altered when bovine blastocysts were cultured in the presence of 3 mM (compared of 0 mM) glucose. Metabolites shown in green decreased and those in red increased in blastocysts exposed to 3 mM glucose, or in medium conditioned by these blastocysts, compared to control conditions (without glucose).

Despite the apparent increase in embryo glycolysis, reduced concentrations of Kreb’s cycle components indicate reduced activity of this important energy producing pathway under hyperglycemic conditions (Fig 4). It is therefore possible that high glucose concentrations result in defective mitochondrial oxidative phosphorylation. In any case, reduced tricarboxylic acid cycle activity indicates inefficient energy production under hyperglycemic conditions. As shown in rats [47] and mice [48], citrate and isocitrate are decreased under diabetic conditions. Similarly, the glucose-treated blastocysts in the current study showed a decrease in citrate and, while isocitrate was not detected in the current study, a decrease in isocitrate is possible, since aconitate and alpha-ketoglutarate were decreased. The decrease in the components in the chain from citrate to alpha -ketoglutarate might be explained by inhibition of the enzyme aconitase by reactive oxygen species [49, 50]; the unchanged levels of the tricarboxylic acid cycle metabolites, malate and fumarate, might be explained by maintenance of the reaction from alpha -ketoglutarate to succinyl-CoA by glutamate [50].

In short, most of the changes detected in glucose-stimulated blastocysts were consistent with altered carbohydrate metabolism, and it appears that these blastocysts use the pentose phosphate pathway preferentially to deal with the elevated glucose concentrations in their local environment.

Glucose can affect the sex ratio of developing embryos, with male embryos developing faster in glucose supplemented medium and female embryos arresting at the morula stage [51]. Possibly, sex ratio modulation is influenced by the pentose phosphate pathway [52], which is also upregulated in the data presented here. It is possible that the analyzed blastocysts were skewed to male blastocysts, but this would also occur in vivo in the female reproductive tract in high glucose conditions.

Furthermore, in mouse preimplantation embryos, epigenetic reprogramming, and in particular DNA methylation and histone modifications, take place at the blastocyst stage [53]. In cattle, it has been demonstrated that male embryos at the blastocyst stage showed a higher DNA methylation compared to female embryos at this stage [54]. Moreover, the nutritional environment to which the early embryo is exposed is able to alter the extent and position of DNA methylations, and can thereby affect the risk of the resulting offspring developing metabolic diseases [55]. In the current study, we expected glucose exposure to lead to altered concentrations of S-adenosylmethionine (SAM), a substrate for methyl-transferases. However, while SAM was detected in cumulus cells (data not shown) it was not detected in blastocysts, irrespective of the glucose content of the culture medium. On the other hand, since entire blastocysts were examined, and differences in DNA methylation are known to exist between trophectoderm and inner cell mass cells, changes in SAM concentrations may have been obscured by differences between the two cell types.

In summary, in this study we describe the effect on metabolic pathways of exposing early bovine embryos to high concentrations of glucose, as would be expected to occur in pregnant diabetic patients. It should be noted that, during diabetes, other tissues/cells including those of the oviduct and uterus will also be exposed to high glucose concentrations which may alter their behaviour and secretory activity accordingly and thereby modify effects seen at the level of the embryo.

## Supporting information

S1 Table List of biochemical compounds detected in bovine blastocysts. Fold changes in biochemical components were calculated for 3 mM glucose-exposed blastocysts versus 0 mM (control) blastocysts. Cells marked in red indicate a significant increase and those marked in green a significant decrease; *–Imputation was used to calculate the fold changes, because the component was not detected in one sample group; #–levels were at around the limit of detection. KEGG—Kyoto Encyclopedia of Genes and Genomes Identifier; PUBCHEM—PubChem Compound Identifier; HMDB—Human Metabolon Data Base Identifier.
(PDF)

S2 Table List of biochemical compounds detected in d5 –d8 medium conditioned by developing bovine embryos during days 5–8 of *in vitro* production. Fold changes in biochemical components were calculated for embryo-conditioned 3 mM glucose supplemented medium versus 0 mM (control) embryo-conditioned medium. Cells marked in red indicate a significant increase and those marked in green a significant decrease; *–Imputation was used to calculate the fold changes, because the component was not detected in one sample group; #–levels were at around the limit of detection. KEGG—Kyoto Encyclopedia of Genes and Genomes Identifier; PUBCHEM—PubChem Compound Identifier; HMDB—Human Metabolon Data Base Identifier.
(PDF)
