# Supplementary material for: Exposure to elevated glucose concentrations alters the metabolomic profile of bovine blastocysts
Source: PLoS One. 2018 Jun 20;13(6):e0199310. doi: 10.1371/journal.pone.0199310 (PMC6010268; doi:10.1371/journal.pone.0199310)
Supplement: S1 Table — Fold changes in biochemical components were calculated for 3 mM glucose-exposed blastocysts versus 0 mM (control) blastocysts. Cells marked in red indicate a significant increase and those marked in green a significant decrease; *–Imputation was used to calculate the fold changes, because the component was not detected in one sample group; #–levels were at around the limit of detection. KEGG—Kyoto Encyclopedia of Genes and Genomes Identifier; PUBCHEM—PubChem Compound Identifier; HMDB—Human Metabolon Data Base Identifier. (PDF) [file pone.0199310.s001.pdf]

Glucose stimulation, Blastocyst

Pathway	Sub Pathway	Biochemical Name	KEGG	PUBCHEM	HMDB	Fold Change Glucose / Control
Amino Acid	Glycine, Serine and Threonine Metabolism	glycine	C00037	750	HMDB00123	0.59
		N-acetylglycine		10972	HMDB00532	0.85
		betaine	C00719	247	HMDB00043	0.50
		serine	C00065	5951	HMDB00187	0.92
		N-acetylserine		65249	HMDB02931	0.83
		threonine	C00188	6288	HMDB00167	0.76
		N-acetylthreonine		152204		0.65
	Alanine and Aspartate Metabolism	alanine	C00041	5950	HMDB00161	0.66
		N-acetylalanine	C02847	88064	HMDB00766	0.69
		aspartate	C00049	5960	HMDB00191	1.20
		N-acetylaspartate (NAA)	C01042	65065	HMDB00812	0.54
		asparagine	C00152	6267	HMDB00168	0.74
	Glutamate Metabolism	glutamate	C00025	611	HMDB00148	0.99
		glutamine	C00064	5961	HMDB00641	0.74
		N-acetylglutamate	C00624	70914	HMDB01138	0.40
		N-acetylglutamine	C02716	182230	HMDB06029	0.39
		pyroglutamine		134508		0.45
		N-acetyl-aspartyl-glutamate (NAAG)	C12270	5255	HMDB01067	0.40
		beta-citrylglutamate	C20775	72715786		0.46
	Histidine Metabolism	histidine	C00135	6274	HMDB00177	0.76
	Lysine Metabolism	lysine	C00047	5962	HMDB00182	0.63
		N6,N6,N6-trimethyllysine	C03793	440120	HMDB01325	0.31
		2-aminoadipate	C00956	469	HMDB00510	0.17
	Phenylalanine Metabolism	phenylalanine	C00079	6140	HMDB00159	0.79
	Tyrosine Metabolism	tyrosine	C00082	6057	HMDB00158	0.71
		3-(4-hydroxyphenyl)lactate	C03672	9378	HMDB00755	0.22
		phenol sulfate	C02180	74426	HMDB60015	0.20
	Tryptophan Metabolism	tryptophan	C00078	6305	HMDB00929	0.87
	Leucine, Isoleucine and Valine Metabolism	leucine	C00123	6106	HMDB00687	0.86
		4-methyl-2-oxopentanoate	C00233	70	HMDB00695	0.51
		beta-hydroxyisovalerate		69362	HMDB00754	0.37
		isoleucine	C00407	6306	HMDB00172	0.79
		3-methyl-2-oxovalerate	C00671	47	HMDB03736	0.39
		methylsuccinate		10349	HMDB01844	0.72
		valine	C00183	6287	HMDB00883	0.81
		3-methyl-2-oxobutyrate	C00141	49	HMDB00019	0.53
	Methionine, Cysteine, SAM and Taurine Metabolism	methionine	C00073	6137	HMDB00696	0.78
		N-acetylmethionine	C02712	448580	HMDB11745	0.80
		methionine sulfoxide	C02989	158980	HMDB02005	0.79
		cysteine	C00097	5862	HMDB00574	0.78
		hypotaurine	C00519	107812	HMDB00965	0.19
	Urea cycle; Arginine and Proline Metabolism	arginine	C00062	232	HMDB00517	0.70
		urea	C00086	1176	HMDB00294	0.59
		ornithine	C00077	6262	HMDB03374	0.48
		proline	C00148	145742	HMDB00162	0.80
		dimethylarginine (SDMA + ADMA)	C03626	123831	HMDB01539	0.31
		trans-4-hydroxyproline	C01157	5810	HMDB00725	0.86
	Creatine Metabolism	creatine	C00300	586	HMDB00064	0.51
		creatinine	C00791	588	HMDB00562	0.82
	Polyamine Metabolism	putrescine	C00134	1045	HMDB01414	0.30
		spermidine	C00315	1102	HMDB01257	0.99
		5-methylthioadenosine (MTA)	C00170	439176	HMDB01173	0.91
		N-acetylputrescine	C02714	122356	HMDB02064	0.14
	Guanidino and Acetamido Metabolism	4-guanidinobutanoate	C01035	500	HMDB03464	0.29
	Glutathione Metabolism	glutathione, oxidized (GSSG)	C00127	65359	HMDB03337	0.21
		5-oxoproline	C01879	7405	HMDB00267	0.85
Peptide	Gamma-glutamyl Amino Acid	gamma-glutamylglutamine	C05283	150914	HMDB11738	0.32
		gamma-glutamylthreonine		76078708	HMDB29159	0.63
	Dipeptide	phenylalanylglycine		98207	HMDB28995	0.52
	Acetylated Peptides	phenylacetylglycine	C05598	68144	HMDB00821	0.82
Carbohydrate	Glycolysis, Gluconeogenesis, and Pyruvate Metabolism	glucose	C00031	79025	HMDB00122	54.19
		3-phosphoglycerate	C00597	724	HMDB00807	11.89
		phosphoenolpyruvate (PEP)	C00074	1005	HMDB00263	4.19
		pyruvate	C00022	1060	HMDB00243	1.09
		lactate	C00186	612	HMDB00190	1.08
		glycerate	C00258	752	HMDB00139	2.42
	Pentose Metabolism	ribose	C00121	5779	HMDB00283	0.55
		sedoheptulose		5459879	HMDB03219	24.99
	Glycogen Metabolism	maltotetraose	C02052	446495	HMDB01296	7.18
		maltotriose	C01835	439586	HMDB01262	13.49
		maltose	C00208	10991489	HMDB00163	21.53
	Fructose, Mannose and Galactose Metabolism	fructose	C00095	5984	HMDB00660	63.04
		mannitol/sorbitol	C00794	5780	HMDB00247	308.98
	Nucleotide Sugar	UDP-glucose	C00029	8629	HMDB00286	60.06
		UDP-galactose	C00052	18068	HMDB00302	31.83
		UDP-N-acetylglucosamine/galactosamine				26.68
	Aminosugar Metabolism	glucuronate	C00191	444791	HMDB00127	0.70
		N-acetyl-glucosamine 1-phosphate	C04256	440364	HMDB01367	9.81
		N-acetylneuraminate	C00270	439197	HMDB00230	3.01
		erythronate		2781043	HMDB00613	1.97
		N-acetylglucosamine/N-acetylgalactosamine		24139	HMDB00215	2.45
		citrate	C00158	311	HMDB00094	0.35

Energy	TCA Cycle	aconitate [cis or trans]				0.38
		alpha-ketoglutarate	C00026	51	HMDB00208	0.44
		succinate	C00042	1110	HMDB00254	0.84
		fumarate	C00122	444972	HMDB00134	1.08
		malate	C00149	525	HMDB00156	1.02
	Oxidative Phosphorylation	acetylphosphate	C00227	186	HMDB01494	3.42
		phosphate	C00009	1061	HMDB01429	0.95
Lipid	Medium Chain Fatty Acid	caprylate (8:0)	C06423	379	HMDB00482	0.75
	Long Chain Fatty Acid	myristoleate (14:1n5)	C08322	5281119	HMDB02000	1.18
		palmitate (16:0)	C00249	985	HMDB00220	1.23
		palmitoleate (16:1n7)	C08362	445638	HMDB03229	1.23
		10-heptadecenoate (17:1n7)		5312435	HMDB60038	1.52
		oleate/vaccenate (18:1)				1.29
		10-nonadecenoate (19:1n9)		5312513	HMDB13622	1.00
		eicosenoate (20:1)	C16526	5282768	HMDB02231	0.95
		erucate (22:1n9)	C08316	5281116	HMDB02068	1.53
		nervonate (24:1n9)	C08323	5281120	HMDB02368	1.02
	Polyunsaturated Fatty Acid (n3 and n6)	eicosapentaenoate (EPA; 20:5n3)	C06428	446284	HMDB01999	0.85
		docosapentaenoate (n3 DPA; 22:5n3)	C16513	6441454	HMDB06528	0.98
		docosahexaenoate (DHA; 22:6n3)	C06429	445580	HMDB02183	0.32
		linoleate (18:2n6)	C01595	5280450	HMDB00673	4.14
		linolenate [alpha or gamma; (18:3n3 or 6)]	C06426	5280934	HMDB03073	1.36
		dihomo-linolenate (20:3n3 or n6)	C03242	5280581	HMDB02925	0.87
		arachidonate (20:4n6)	C00219	444899	HMDB01043	0.82
		docosadienoate (22:2n6)	C16533	5282807	HMDB61714	1.35
		dihomo-linoleate (20:2n6)	C16525	6439848	HMDB05060	1.18
		mead acid (20:3n9)		5312531	HMDB10378	0.73
	Fatty Acid, Dicarboxylate	glutarate (pentanedioate)	C00489	743	HMDB00661	0.46
		2-hydroxyglutarate	C02630	43	HMDB00606	0.73
		maleate	C01384	444266	HMDB00176	0.94
	Fatty Acid Metabolism (also BCAA Metabolism)	propionylcarnitine (C3)	C03017	107738	HMDB00824	0.25
		methylmalonate (MMA)	C02170	487	HMDB00202	5.54
	Fatty Acid Metabolism(Acyl Carnitine)	stearoylcarnitine (C18)		6426855	HMDB00848	0.23
	Fatty Acid, Monohydroxy	2-hydroxypalmitate		92836	HMDB31057	0.95
		3-hydroxyhexanoate		151492		0.75
		3-hydroxyoctanoate		26613	HMDB01954	1.03
		13-HODE + 9-HODE		43013		0.84
	Inositol Metabolism	myo-inositol	C00137	892	HMDB00211	3.47
	Phospholipid Metabolism	choline	C00114	305	HMDB00097	0.61
		choline phosphate	C00588	1014	HMDB01565	0.68
		glycerophosphorylcholine (GPC)	C00670	71920	HMDB00086	1.01
		phosphoethanolamine	C00346	1015	HMDB00224	0.39
		glycerophosphoethanolamine	C01233	123874	HMDB00114	1.05
		glycerophosphoinositol		167572		1.72
	Phosphatidylcholine (PC)	1-myristoyl-2-palmitoyl-GPC (14:0/16:0)		129657	HMDB07869	0.99
		1,2-dipalmitoyl-GPC (16:0/16:0)	D03585	452110	HMDB00564	0.92
		1-palmitoyl-2-palmitoleoyl-GPC (16:0/16:1)			HMDB07969	1.01
		1-palmitoyl-2-stearoyl-GPC (16:0/18:0)			HMDB07970	1.12
		1-palmitoyl-2-oleoyl-GPC (16:0/18:1)		6436017	HMDB07972	0.96
		1-palmitoyl-2-linoleoyl-GPC (16:0/18:2)		5287971	HMDB07973	0.91
		1-palmitoyl-2-alpha-linolenoyl-GPC (16:0/18:3n3)			HMDB07975	0.78
		1-palmitoleoyl-2-linoleoyl-GPC (16:1/18:2)			HMDB08006	1.31
		1-palmitoyl-2-arachidonoyl-GPC (16:0/20:4n6)		10747814	HMDB07982	0.79
		1-palmitoyl-2-docosahexaenoyl-GPC (16:0/22:6)*		6441886	HMDB07991	1.00
		1-stearoyl-2-oleoyl-GPC (18:0/18:1)			HMDB08038	1.55
		1,2-dioleoyl-GPC (18:1/18:1)		10350317		1.08
		1-oleoyl-2-linoleoyl-GPC (18:1/18:2)				0.92
		1,2-dilinoleoyl-GPC (18:2/18:2)		5288075	HMDB08138	1.79
		1-stearoyl-2-arachidonoyl-GPC (18:0/20:4)		16219824	HMDB08048	1.23
	Phosphatidylethanolamine (PE)	1-palmitoyl-2-oleoyl-GPE (16:0/18:1)		5283496	HMDB05320	0.90
		1-palmitoyl-2-linoleoyl-GPE (16:0/18:2)		9546747	HMDB05322	0.49
		1-palmitoleoyl-2-oleoyl-GPE (16:1/18:1)				2.09
		1-palmitoyl-2-arachidonoyl-GPE (16:0/20:4)		9546800	HMDB05323	0.81
		1-stearoyl-2-oleoyl-GPE (18:0/18:1)			HMDB08993	1.06
		1,2-dioleoyl-GPE (18:1/18:1)		9546757		1.04
		1-oleoyl-2-linoleoyl-GPE (18:1/18:2)		9546753	HMDB05349	1.07
		1-stearoyl-2-arachidonoyl-GPE (18:0/20:4)		5289133	HMDB09003	1.05
		1-oleoyl-2-arachidonoyl-GPE (18:1/20:4)			HMDB09069	0.88
	Phosphatidylserine (PS)	1-palmitoyl-2-oleoyl-GPS (16:0/18:1)	C13880	5283499	HMDB12357	0.58
		1-stearoyl-2-oleoyl-GPS (18:0/18:1)		9547087	HMDB10163	0.88
	Phosphatidylinositol (PI)	1-palmitoyl-2-arachidonoyl-GPI (16:0/20:4)			HMDB09789	1.25
		1-stearoyl-2-oleoyl-GPI (18:0/18:1)				1.44
		1-stearoyl-2-arachidonoyl-GPI (18:0/20:4)			HMDB09815	0.68
		1-oleoyl-2-arachidonoyl-GPI (18:1/20:4)			HMDB09844	0.93
	Lysophospholipid	1-palmitoyl-GPC (16:0)		86554	HMDB10382	0.88
		2-palmitoyl-GPC (16:0)		15061532	HMDB61702	0.77
		1-stearoyl-GPC (18:0)		497299	HMDB10384	1.35
		1-oleoyl-GPC (18:1)		16081932	HMDB02815	0.72
		1-stearoyl-GPE (18:0)		9547068	HMDB11130	0.89
		1-oleoyl-GPE (18:1)		9547071	HMDB11506	0.71
		1-oleoyl-GPG (18:1)				0.51
		1-stearoyl-GPI (18:0)			HMDB61696	1.91
		1-(1-enyl-palmitoyl)-2-oleoyl-GPE (P-16:0/18:1)			HMDB11342	1.01
		1-(1-enyl-palmitoyl)-2-linoleoyl-GPE (P-16:0/18:2)			HMDB11343	1.20
		1-(1-enyl-palmitoyl)-2-palmitoyl-GPC (P-16:0/16:0)		11146967	HMDB11206	0.63

	Plasmalogen	1-(1-enyl-palmitoyl)-2-palmitoleoyl-GPC (P-16:0/16:1)			HMDB11207	0.27
		1-(1-enyl-palmitoyl)-2-arachidonoyl-GPE (P-16:0/20:4)			HMDB11352	0.97
		1-(1-enyl-palmitoyl)-2-oleoyl-GPC (P-16:0/18:1)				0.74
		1-(1-enyl-stearoyl)-2-oleoyl-GPE (P-18:0/18:1)			HMDB11375	0.86
		1-(1-enyl-palmitoyl)-2-arachidonoyl-GPC (P-16:0/20:4)			HMDB11220	0.47
		1-(1-enyl-palmitoyl)-2-linoleoyl-GPC (P-16:0/18:2)			HMDB11211	0.85
		1-(1-enyl-stearoyl)-2-arachidonoyl-GPE (P-18:0/20:4)		9547058	HMDB05779	0.90
	Lysoplasmalogen	1-(1-enyl-oleoyl)-2-oleoyl-GPE (P-18:1/18:1)			HMDB11441	1.05
	Glycerolipid Metabolism	glycerol	C00116	753	HMDB00131	1.15
		glycerol 3-phosphate	C00093	754	HMDB00126	12.72
		glycerophosphoglycerol	C03274	439964		1.71
	Monoacylglycerol	1-pentadecanoylglycerol (15:0)		190750		1.27
		1-palmitoylglycerol (16:0)		14900	HMDB31074	1.63
		1-palmitoleoylglycerol (16:1)			HMDB11565	1.82
		1-linoleoylglycerol (18:2)		5283469		1.40
		2-myristoylglycerol (14:0)		137938	HMDB11530	2.31
		2-palmitoylglycerol (16:0)		123409	HMDB11533	0.36
	Diacylglycerol	diacylglycerol (12:0/18:1, 14:0/16:1, 16:0/14:1) [2]				1.75
		diacylglycerol (14:0/18:1, 16:0/16:1) [1]				0.84
		diacylglycerol (14:0/18:1, 16:0/16:1) [2]				1.28
		diacylglycerol (16:1/18:2 [2], 16:0/18:3 [1])				0.76
		palmitoyl-myristoyl-glycerol (16:0/14:0) [2]			HMDB07095	1.47
		palmitoyl-palmitoyl-glycerol (16:0/16:0) [1]			HMDB07098	1.17
		palmitoyl-palmitoyl-glycerol (16:0/16:0) [2]			HMDB07098	0.93
		palmitoyl-oleoyl-glycerol (16:0/18:1) [1]	C13861		HMDB07102	0.90
		palmitoyl-oleoyl-glycerol (16:0/18:1) [2]	C13861		HMDB07102	0.95
		palmitoyl-linoleoyl-glycerol (16:0/18:2) [2]			HMDB07103	1.43
		palmitoleoyl-oleoyl-glycerol (16:1/18:1) [2]				2.09
		palmitoleoyl-linoleoyl-glycerol (16:1/18:2) [1]			HMDB07132	1.07
		palmitoyl-dihomo-linolenoyl-glycerol (16:0/20:3n3 or 6) [2]				0.97
		palmitoyl-arachidonoyl-glycerol (16:0/20:4) [2]			HMDB07112	1.66
		oleoyl-oleoyl-glycerol (18:1/18:1) [1]			HMDB07218	0.97
		oleoyl-oleoyl-glycerol (18:1/18:1) [2]			HMDB07218	1.09
		oleoyl-linoleoyl-glycerol (18:1/18:2) [2]			HMDB07219	1.07
		linoleoyl-linoleoyl-glycerol (18:2/18:2) [1]			HMDB07248	0.99
		stearoyl-arachidonoyl-glycerol (18:0/20:4) [1]				0.96
		stearoyl-arachidonoyl-glycerol (18:0/20:4) [2]				1.05
		oleoyl-arachidonoyl-glycerol (18:1/20:4) [1]*			HMDB07228	1.00
		oleoyl-arachidonoyl-glycerol (18:1/20:4) [2]			HMDB07228	1.01
	Sphingolipid Metabolism	sphinganine	C00836	3126	HMDB00269	2.26
		N-palmitoyl-sphinganine (d18:0/16:0)		5283572	HMDB11760	1.01
		N-palmitoyl-sphingadienine (d18:2/16:0)				0.35
		palmitoyl dihydrosphingomyelin (d18:0/16:0)		9939965		0.82
		palmitoyl sphingomyelin (d18:1/16:0)		9939941		0.69
		stearoyl sphingomyelin (d18:1/18:0)	C00550	6453725	HMDB01348	0.83
		behenoyl sphingomyelin (d18:1/22:0)			HMDB12103	0.92
		lignoceroyl sphingomyelin (d18:1/24:0)				1.00
		sphingomyelin (d18:1/14:0, d16:1/16:0)		11433862	HMDB12097	0.93
		sphingomyelin (d17:1/16:0, d18:1/15:0, d16:1/17:0)				0.62
		sphingomyelin (d18:2/16:0, d18:1/16:1)				0.42
		sphingomyelin (d18:1/17:0, d17:1/18:0, d19:1/16:0)				0.89
		sphingomyelin (d18:1/18:1, d18:2/18:0)		6443882	HMDB12101	0.70
		sphingomyelin (d18:1/20:0, d16:1/22:0)			HMDB12102	1.17
		sphingomyelin (d18:1/24:1, d18:2/24:0)			HMDB12107	0.85
		sphingomyelin (d18:2/24:1, d18:1/24:2)				0.74
		sphingosine	C00319	5353955	HMDB00252	1.07
		N-palmitoyl-heptadecasphingosine (d17:1/16:0)				0.61
	Ceramides	N-palmitoyl-sphingosine (d18:1/16:0)		5283564	HMDB04949	0.82
		N-stearoyl-sphingosine (d18:1/18:0)		5283565	HMDB04950	0.87
		ceramide (d16:1/24:1, d18:1/22:1)*				1.00
		ceramide (d18:1/17:0, d17:1/18:0)				0.88
		ceramide (d18:2/24:1, d18:1/24:2)				0.61
		glycosyl-N-palmitoyl-sphingosine (d18:1/16:0)				1.15
		glycosyl-N-stearoyl-sphingosine (d18:1/18:0)				1.26
		lactosyl-N-palmitoyl-sphingosine (d18:1/16:0)				0.80
	Mevalonate Metabolism	3-hydroxy-3-methylglutarate	C03761	1662	HMDB00355	0.57
	Sterol	cholesterol	C00187	11025495	HMDB00067	0.89
Nucleotide	Purine Metabolism, (Hypo)Xanthine/Inosine containing	inosine	C00294	6021	HMDB00195	0.82
		hypoxanthine	C00262	790	HMDB00157	0.88
		xanthine	C00385	1188	HMDB00292	0.78
	Purine Metabolism, Adenine containing	adenosine 5'-monophosphate (AMP)	C00020	6083	HMDB00045	2.23
		adenosine 3'-monophosphate (3'-AMP)	C01367	41211	HMDB03540	1.00
		adenosine	C00212	60961	HMDB00050	1.11
		adenine	C00147	190	HMDB00034	1.33
	Purine Metabolism, Guanine containing	guanosine	C00387	6802	HMDB00133	0.79
		guanine	C00242	764	HMDB00132	0.71
	Pyrimidine Metabolism, Orotate containing	orotate	C00295	967	HMDB00226	1.31
	Pyrimidine Metabolism, Uracil containing	uridine	C00299	6029	HMDB00296	0.80
		pseudouridine	C02067	15047	HMDB00767	0.48
		2'-deoxyuridine	C00526	13712	HMDB00012	0.52
		beta-alanine	C00099	239	HMDB00056	0.32
	Pyrimidine Metabolism, Cytidine containing	cytidine 5'-monophosphate (5'-CMP)	C00055	6131	HMDB00095	2.20
		cytidine	C00475	6175	HMDB00089	0.65
		2'-deoxycytidine	C00881	13711	HMDB00014	0.97
	Pyrimidine Metabolism, Thymine containing	thymidine	C00214	5789	HMDB00273	0.24

Cofactors and Vitamins	Nicotinate and Nicotinamide Metabolism	nicotinamide	C00153	936	HMDB01406	1.08
	Pantothenate and CoA Metabolism	pantothenate	C00864	6613	HMDB00210	1.12
	Ascorbate and Aldarate Metabolism	gulonate	C00257	9794176	HMDB03290	0.85
	Tocopherol Metabolism	alpha-tocopherol	C02477	14985	HMDB01893	0.82
Xenobiotics	Benzoate Metabolism	benzoate	C00180	243	HMDB01870	0.17
		p-cresol sulfate		4615423	HMDB11635	0.84
	Food Component/Plant	gluconate	C00257	10690	HMDB00625	1.94
		beta-guanidinopropanoate	C03065	67701	HMDB13222	1.00
		erythritol	C00503	222285	HMDB02994	1.98
	Drug	penicillin G	C05551	5904	HMDB15186	0.84
		salicylate	C00805	338	HMDB01895	0.70
	Chemical	sulfate	C00059	1118	HMDB01448	0.94
		HEPES		23831		0.21
		phenol red	C12600	4766		0.86
		trizma acetate	C07182	6503		0.70
		thioproline		93176		0.76
