# Supplementary material for: Exposure to elevated glucose concentrations alters the metabolomic profile of bovine blastocysts
Source: PLoS One. 2018 Jun 20;13(6):e0199310. doi: 10.1371/journal.pone.0199310 (PMC6010268; doi:10.1371/journal.pone.0199310)
Supplement: S2 Table — Fold changes in biochemical components were calculated for embryo-conditioned 3 mM glucose supplemented medium versus 0 mM (control) embryo-conditioned medium. Cells marked in red indicate a significant increase and those marked in green a significant decrease; *–Imputation was used to calculate the fold changes, because the component was not detected in one sample group; #–levels were at around the limit of detection. KEGG—Kyoto Encyclopedia of Genes and Genomes Identifier; PUBCHEM—PubChem Compound Identifier; HMDB—Human Metabolon Data Base Identifier. (PDF) [file pone.0199310.s002.pdf]

**Glucose stimulation, medium**

| Pathway | Sub Pathway | Biochemical Name | KEGG | PUBCHEM | HMDB | Fold Change Glucose / Control |
|---|---|---|---|---|---|---|
| Amino Acid | Glycine, Serine and Threonine Metabolism | glycine | C00037 | 750 | HMDB00123 | 0.96 |
| | | betaine | C00719 | 247 | HMDB00043 | 0.92 |
| | | serine | C00065 | 5951 | HMDB00187 | 0.96 |
| | | N-acetylserine | | 65249 | HMDB02931 | 1.04 |
| | | threonine | C00188 | 6288 | HMDB00167 | 0.91 |
| | | N-acetylthreonine | | 152204 | | 1.16 |
| | Alanine and Aspartate Metabolism | alanine | C00041 | 5950 | HMDB00161 | 0.96 |
| | | N-acetylalanine | C02847 | 88064 | HMDB00766 | 1.15 |
| | | aspartate | C00049 | 5960 | HMDB00191 | 0.93 |
| | | N-acetylaspartate (NAA) | C01042 | 65065 | HMDB00812 | 1.09 |
| | | asparagine | C00152 | 6267 | HMDB00168 | 0.93 |
| | | N-acetylasparagine | | 99715 | HMDB06028 | 1.69 |
| | Glutamate Metabolism | glutamate | C00025 | 611 | HMDB00148 | 0.96 |
| | | glutamine | C00064 | 5961 | HMDB00641 | 0.97 |
| | | N-acetylglutamate | C00624 | 70914 | HMDB01138 | 1.57 |
| | | N-acetylglutamine | C02716 | 182230 | HMDB06029 | 1.21 |
| | | pyroglutamine | | 134508 | | 0.98 |
| | Histidine Metabolism | histidine | C00135 | 6274 | HMDB00177 | 0.93 |
| | Lysine Metabolism | lysine | C00047 | 5962 | HMDB00182 | 0.91 |
| | Phenylalanine Metabolism | phenylalanine | C00079 | 6140 | HMDB00159 | 0.95 |
| | | N-acetylphenylalanine | C03519 | 74839 | HMDB00512 | 1.37 |
| | | phenylpyruvate | C00166 | 997 | HMDB00205 | 0.62 |
| | Tyrosine Metabolism | tyrosine | C00082 | 6057 | HMDB00158 | 0.92 |
| | | phenol sulfate | C02180 | 74426 | HMDB60015 | 1.08 |
| | | o-Tyrosine | | 91482 | HMDB06050 | 1.06 |
| | | N-formylphenylalanine | | 759256 | | 0.98 |
| | Tryptophan Metabolism | tryptophan | C00078 | 6305 | HMDB00929 | 0.94 |
| | Leucine, Isoleucine and Valine Metabolism | leucine | C00123 | 6106 | HMDB00687 | 0.96 |
| | | N-acetylleucine | C02710 | 70912 | HMDB11756 | 1.10 |
| | | 4-methyl-2-oxopentanoate | C00233 | 70 | HMDB00695 | 0.66 |
| | | isovalerylglycine | | 546304 | HMDB00678 | 1.03 |
| | | beta-hydroxyisovalerate | | 69362 | HMDB00754 | 0.65 |
| | | isoleucine | C00407 | 6306 | HMDB00172 | 0.98 |
| | | 3-methyl-2-oxovalerate | C00671 | 47 | HMDB03736 | 0.63 |
| | | valine | C00183 | 6287 | HMDB00883 | 0.93 |
| | | 3-methyl-2-oxobutyrate | C00141 | 49 | HMDB00019 | 0.73 |
| | | 3-hydroxyisobutyrate | C06001 | 87 | HMDB00336 | 0.70 |
| | Methionine, Cysteine, SAM and Taurine Metabolism | methionine | C00073 | 6137 | HMDB00696 | 0.93 |
| | | N-acetylmethionine* | C02712 | 448580 | HMDB11745 | 1.00 |
| | | methionine sulfoxide | C02989 | 158980 | HMDB02005 | 1.43 |
| | | cysteine | C00097 | 5862 | HMDB00574 | 0.90 |
| | | cysteine s-sulfate | C05824 | 115015 | HMDB00731 | 0.96 |
| | | cystine | C00491 | 67678 | HMDB00192 | 1.00 |
| | | cysteine sulfinic acid | C00606 | 109 | HMDB00996 | 1.61 |
| | | 3-sulfo-L-alanine | C00506 | 72886 | HMDB02757 | 1.00 |
| | Urea cycle; Arginine and Proline Metabolism | arginine | C00062 | 232 | HMDB00517 | 0.97 |
| | | urea | C00086 | 1176 | HMDB00294 | 0.40 |
| | | ornithine | C00077 | 6262 | HMDB03374 | 0.91 |
| | | 2-oxoarginine* | C03771 | 558 | HMDB04225 | 1.00 |
| | | proline | C00148 | 145742 | HMDB00162 | 0.95 |
| | | dimethylarginine (SDMA + ADMA) | C03626 | 123831 | HMDB01539 | 0.94 |
| | | N-acetylproline* | | 322640 | | 1.00 |
| | | trans-4-hydroxyproline | C01157 | 5810 | HMDB00725 | 1.43 |
| | Creatine Metabolism | creatine* | C00300 | 586 | HMDB00064 | 1.00 |
| | | creatinine | C00791 | 588 | HMDB00562 | 1.02 |
| | Guanidino and Acetamido Metabolism | 4-guanidinobutanoate | C01035 | 500 | HMDB03464 | 0.55 |
| | Glutathione Metabolism | 5-oxoproline | C01879 | 7405 | HMDB00267 | 0.93 |
| | | 2-hydroxybutyrate/2-hydroxyisobutyrate | | | | 1.09 |
| Peptide | Gamma-glutamyl Amino Acid | gamma-glutamylglutamine | C05283 | 150914 | HMDB11738 | 0.90 |
| | | gamma-glutamylisoleucine | | 14253342 | HMDB11170 | 0.78 |
| | | gamma-glutamylleucine | | 151023 | HMDB11171 | 0.93 |
| | | gamma-glutamylvaline | | 7015683 | HMDB11172 | 0.93 |
| | Acetylated Peptides | phenylacetylglycine | C05598 | 68144 | HMDB00821 | 0.96 |
| | Glycolysis, Gluconeogenesis, and Pyruvate Metabolism | glucose | C00031 | 79025 | HMDB00122 | 1922.07 |
| | | pyruvate | C00022 | 1060 | HMDB00243 | 1.42 |
| | | lactate | C00186 | 612 | HMDB00190 | 0.94 |
| | | glycerate | C00258 | 752 | HMDB00139 | 1.15 |
| | | ribitol | C00474 | 6912 | HMDB00508 | 1.01 |

| | | | | | | |
|---|---|---|---|---|---|---|
| Carbohydrate | Pentose Metabolism | ribonate | C01685 | 5460677 | HMDB00867 | 2.46 |
| | | arabonate/xylonate | | | | 2.48 |
| | Glycogen Metabolism | maltose | C00208 | 10991489 | HMDB00163 | 1.10 |
| | Fructose, Mannose and Galactose Metabolism | fructose | C00095 | 5984 | HMDB00660 | 97.73 |
| | | mannitol/sorbitol | C00794 | 5780 | HMDB00247 | 5.83 |
| | | mannose | C00159 | 18950 | HMDB00169 | 1.82 |
| | Aminosugar Metabolism | erythronate | | 2781043 | HMDB00613 | 1.48 |
| | | N-acetylglucosamine/N-acetylgalactosamine | | 24139 | HMDB00215 | 0.63 |
| Energy | TCA Cycle | citrate | C00158 | 311 | HMDB00094 | 0.71 |
| | | aconitate [cis or trans] | | | | 0.96 |
| | | alpha-ketoglutarate | C00026 | 51 | HMDB00208 | 1.13 |
| | | succinate | C00042 | 1110 | HMDB00254 | 0.68 |
| | | fumarate | C00122 | 444972 | HMDB00134 | 1.05 |
| | | malate | C00149 | 525 | HMDB00156 | 1.25 |
| | Oxidative Phosphorylation | phosphate | C00009 | 1061 | HMDB01429 | 1.04 |
| Lipid | Medium Chain Fatty Acid | caproate (6:0) | C01585 | 8892 | HMDB00535 | 1.52 |
| | | heptanoate (7:0) | C17714 | 8094 | HMDB00666 | 0.76 |
| | | caprylate (8:0) | C06423 | 379 | HMDB00482 | 0.98 |
| | Long Chain Fatty Acid | erucate (22:1n9) | C08316 | 5281116 | HMDB02068 | 0.84 |
| | Polyunsaturated Fatty Acid (n3 and n6) | linolenate [alpha or gamma; (18:3n3 or 6)] | C06426 | 5280934 | HMDB03073 | 0.76 |
| | Fatty Acid, Dicarboxylate | glutarate (pentanedioate) | C00489 | 743 | HMDB00661 | 1.05 |
| | | 2-hydroxyglutarate | C02630 | 43 | HMDB00606 | 0.81 |
| | | maleate | C01384 | 444266 | HMDB00176 | 0.94 |
| | | azelate (nonanedioate) | C08261 | 2266 | HMDB00784 | 0.94 |
| | Fatty Acid, Monohydroxy | 3-hydroxyhexanoate | | 151492 | | 1.03 |
| | | 3-hydroxyoctanoate | | 26613 | HMDB01954 | 0.85 |
| | Inositol Metabolism | myo-inositol | C00137 | 892 | HMDB00211 | 2.84 |
| | Phospholipid Metabolism | choline | C00114 | 305 | HMDB00097 | 0.94 |
| | | choline phosphate* | C00588 | 1014 | HMDB01565 | 1.00 |
| | Phosphatidylcholine (PC) | 1-palmitoyl-2-oleoyl-GPC (16:0/18:1) | | 6436017 | HMDB07972 | 0.92 |
| | Glycerolipid Metabolism | glycerol* | C00116 | 753 | HMDB00131 | 1.00 |
| | | glycerophosphoglycerol | C03274 | 439964 | | 1.01 |
| | Mevalonate Metabolism | 3-hydroxy-3-methylglutarate | C03761 | 1662 | HMDB00355 | 0.82 |
| Nucleotide | Purine Metabolism, (Hypo)Xanthine/Inosine containing | inosine | C00294 | 6021 | HMDB00195 | 1.50 |
| | | hypoxanthine | C00262 | 790 | HMDB00157 | 0.77 |
| | | xanthine | C00385 | 1188 | HMDB00292 | 0.70 |
| | Purine Metabolism, Adenine containing | adenine | C00147 | 190 | HMDB00034 | 0.72 |
| | Purine Metabolism, Guanine containing | guanine | C00242 | 764 | HMDB00132 | 0.47 |
| | Pyrimidine Metabolism, Orotate containing | orotate | C00295 | 967 | HMDB00226 | 1.11 |
| | Pyrimidine Metabolism, Uracil containing | uridine | C00299 | 6029 | HMDB00296 | 1.56 |
| | | pseudouridine | C02067 | 15047 | HMDB00767 | 0.83 |
| | | 2'-deoxyuridine* | C00526 | 13712 | HMDB00012 | 1.00 |
| | Pyrimidine Metabolism, Cytidine containing | cytidine | C00475 | 6175 | HMDB00089 | 1.00 |
| | | 2'-deoxycytidine | C00881 | 13711 | HMDB00014 | 1.49 |
| | Pyrimidine Metabolism, Thymine containing | thymidine | C00214 | 5789 | HMDB00273 | 0.90 |
| | Nicotinate and Nicotinamide Metabolism | nicotinamide | C00153 | 936 | HMDB01406 | 0.77 |
| | Pantothenate and CoA Metabolism | pantothenate | C00864 | 6613 | HMDB00210 | 1.10 |
| | Ascorbate and Aldarate Metabolism | threonate | C01620 | 151152 | HMDB00943 | 0.63 |
| | | gulonate* | C00257 | 9794176 | HMDB03290 | 1.00 |
| | Tocopherol Metabolism | alpha-tocopherol | C02477 | 14985 | HMDB01893 | 0.80 |
| | Benzoate Metabolism | benzoate | C00180 | 243 | HMDB01870 | 1.21 |
| | | p-cresol sulfate | | 4615423 | HMDB11635 | 0.89 |
| | Food Component/Plant | maltol | C11918 | 8369 | HMDB30776 | 1.05 |
| | | gluconate | C00257 | 10690 | HMDB00625 | 1.97 |
| | | erythritol* | C00503 | 222285 | HMDB02994 | 1.00 |
| | | stachydrine | C10172 | 115244 | HMDB04827 | 0.71 |
| | Drug | streptomycin | | 5999 | | 0.80 |
| | | penicillin G | C05551 | 5904 | HMDB15186 | 0.09 |
| | | salicylate | C00805 | 338 | HMDB01895 | 1.07 |
| | Chemical | sulfate | C00059 | 1118 | HMDB01448 | 1.00 |
| | | HEPES | | 23831 | | 1.05 |
| | | phenol red | C12600 | 4766 | | 0.92 |
| | | thioproline | | 93176 | | 1.13 |
